# Mercury use on the artisanal gold mining site at Gnikpière, Burkina Faso

**DOI:** 10.1007/s10661-026-16131-1

**Published:** 2026-11-14

**Authors:** Alice Rayim Wendé Naré, Issa Ouédraogo, Moussa Bouboucari

**Affiliations:** 1 Centre National de la Recherche Scientifique et Technologique, Institut de Recherche en Sciences Appliquées et Technologies (IRSAT), Département Substances Naturelles, Ouagadougou, Burkina Faso; 2 Laboratoire Géosciences et Environnement (LaGE), Université Joseph KI-ZERBO, Ouagadougou, Burkina Faso; 3 Centre de Recherche et de Formation pour le Développement Industriel ANDAL, Ouagadougou, Burkina Faso

**Keywords:** Gold mining, Mercury, Soil, Water, Burkina Faso

## Abstract

The general objective of the study is to assess users' knowledge, attitudes, and practices regarding mercury use at the Gnikpière site, and evaluate associated soil and water contamination. A survey was conducted with 40 miners. Twenty-five samples, including 23 soil samples and 2 water samples, were taken to assess their mercury content. Total mercury (THg) analysis of samples was performed using atomic absorption spectroscopy (AAS) after digestion with HNO_3_ and pyrolysis. Hundred percent of the artisanal gold miners mix the gravity concentrate with their hands without protection. For amalgam burning, the most commonly used energy sources are the furnace (35%), gas (10%), a fire pit (10%), or a combination of the mentioned methods (40%). The amalgamating and burning are done at the same site in open areas by 67% of miners, and only 5% of artisanal miners use adequate equipment. The soils at the mills record the highest levels of mercury (138.45 ppm), followed by the washing sludge (52.6 ppm), gold buying sheds (48.15 ppm), washing sheds (19.27 ppm) and finally those from the fields downstream which have the lowest mercury levels (3.65 ppm). In the water samples, the mercury content is below the detection limit. A great proportion of the population of the Gnikpière site claim to be aware of the risks of mercury on their health (87.5%) and on the environment (75%), but only 42.5% have been made aware of the real risks of mercury. In the Gnikpière site, the use of mercury is not appropriate, leading to environmental contamination.

## Introduction

Artisanal mining supports 20–30 million people in Sub-Saharan Africa (Mulenga et al., 2024). In Burkina Faso, the mining sector has developed in recent decades, providing income for many families, especially in rural regions where alternative employment is scarce (Konkobo & Sawadogo, 2020). Artisanal gold mining involves 1.3 million people across 1500 sites in 2018 (Effigis Geo Solutions, 2018) and produces around 9.5 tons of gold, valued at 232.2 billion FCFA. The miners at the artisanal gold mining use toxic chemicals such as mercury, cyanide, zinc, and nitric acid (Kumar et al., 2013). Numerous studies have been conducted on the use of mercury in artisanal gold mining and its impacts on the environment and human health (Grandjean et al., 2010). The combustion of amalgam for the purpose of gold extraction results in the emission of mercury (Hg) vapor into the surrounding ecosystem, thereby exposing miners and their families to risks associated with Hg exposure (AMAP, UNEP., 2013). On a global scale, it is estimated that artisanal and small-scale gold mining (ASGM) accounts for approximately 40% of the total atmospheric mercury (Hg) emissions (AMAP, UNEP., 2013). Mercury exposure represents a major public health concern because its different chemical forms can affect multiple organ systems. Mercury adversely affects the central nervous system, inducing symptoms such as tremors and cognitive impairments and is particularly neurotoxic to developing brains (Grandjean et al., 2010), as well as renal dysfunction and cardiovascular complications, which contribute to hypertension and cardiac diseases (Obun et al., 2025). However, the diagnosis of mercury-related health effects can be challenging because many clinical manifestations are non-specific and may be as those of other diseases or environmental exposures (ATSDR, 2024). Nonetheless, in countries located within sub-Saharan Africa, the sources of mercury pollution remain largely unregulated (Lassen et al., 2016). The studies’ results reveal elevated Hg levels in ASGM top soils with concentrations reaching as high as 410 mg g^−1^ in Ghana and 8 mg g^−1^ in Senegal. Elevated Hg concentrations in aquatic systems have been consistently documented in surface waters surrounding mining regions in Ghana (28.7 to 420 ng l^−1^) (Donkor et al., 2006), Côte d’Ivoire (6.6 to 53 ng l^−1^), (Mason et al., 2019), Senegal (5.8 to 974 ng l^−1^), and Burkina Faso, where levels can reach up to 21 ng l^−1^ (Dabré et al., 2025). Once introduced into aquatic environments, inorganic mercury can be biotransformed by microorganisms into methylmercury (MeHg), a toxic compound that bioaccumulates and biomagnifies within the trophic web.

Upon deposition in the soil matrix, Hg may undergo volatilization or form complexes with organic matter and minerals, including iron oxides and clay minerals. Understanding the distribution of Hg among soil constituents is therefore crucial for documenting the reactivity of Hg carrier phases, which are susceptible to (bio)reduction and (bio)methylation upon deposition in river sediments. This will help to draw the attention of stakeholders and decision-makers to the use of mercury despite its prohibition.

## Methodology

### Study site

**Fig. 1:**
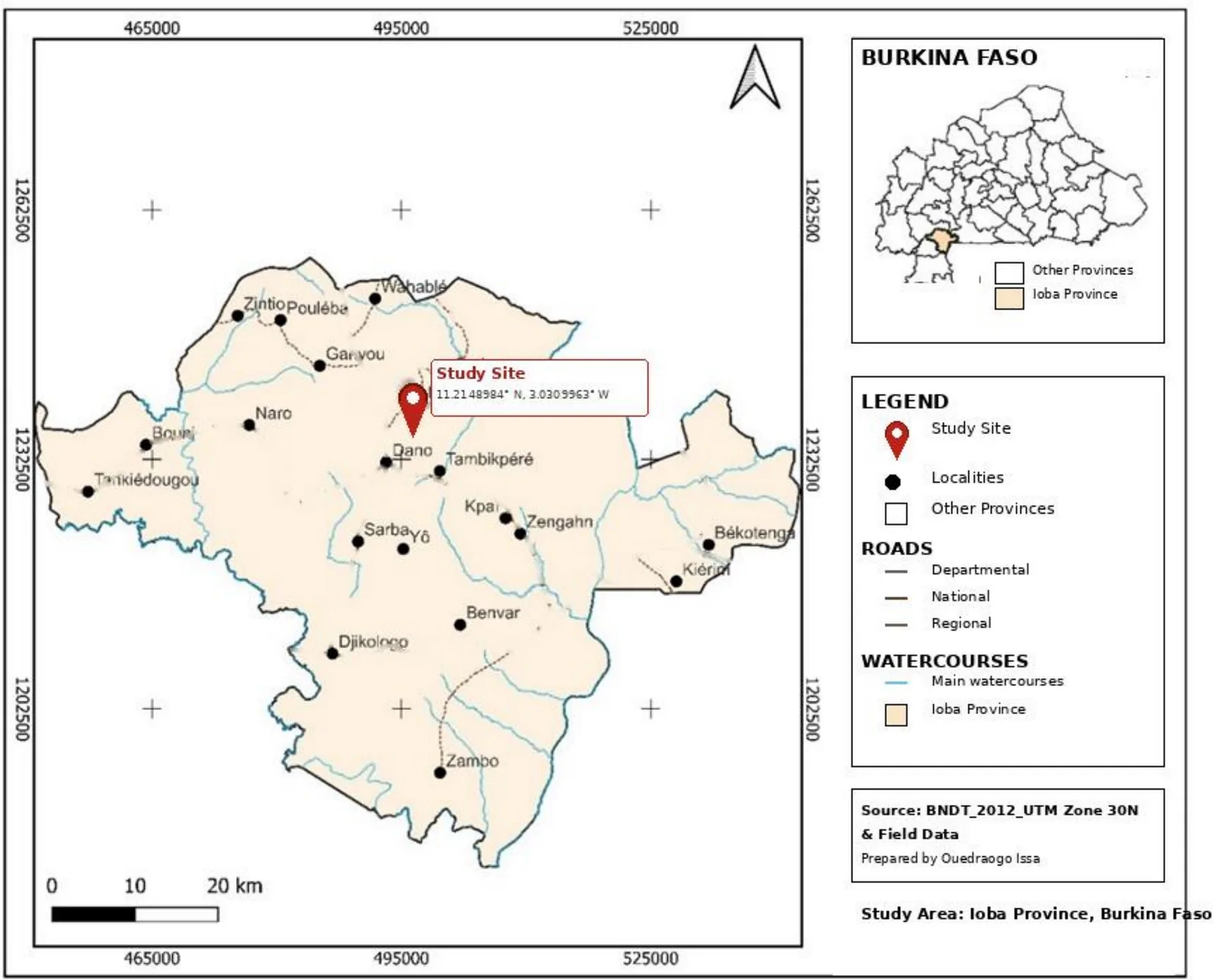
Map of Ioba Province, Burkina Faso, marking the study site near Dano. An inset locates the province within Burkina Faso. The map also identifies localities, roads and main watercourses

The study was conducted in October 2022, at the Gnikpière artisanal gold mining site, located in the village of Gnikpière (Dano administrative department, Djôrô region, Burkina Faso; coordinates: − 3.0309963 E, 11.2148984 N). This region is now a very important area for artisanal gold production, accounting for approximately half of the artisanal gold produced in Burkina Faso in 2016 (INSD, 2017). Survey results from the Artisanal Gold Council (AGC) in 2019 showed that mercury is widely used for gold mining in this region Fig. 1.

### Survey on mercury use

The survey form was developed using the data collection software KoboCollect v2022.1.2. The population selected for the survey was composed of persons who were directly (gold miners) and indirectly (gold buyers) related to gold mining activities. The questionnaire targeted ore washers and primarily focused on the following: the origin of the mercury used; how and the conditions of mercury use; the amount of mercury used; the equipment used; the fate of mercury packaging and waste; users’ perceptions of the dangers associated with mercury use. A survey was conducted randomly among miners who voluntarily agreed to answer the questionnaire and who practice either mercury amalgamation. At the end of the survey, 40 artisanal miners were interviewed at the ore washing sheds, including 25 men and 15 women.

### Estimation of the amount of gold and mercury produced and the mercury/gold ratio

The amount of gold produced per year (Q(Au)/year) at the Gnikpière site was determined by considering the quantity of gold purchased by each gold buyer and their respective number of working days. Indeed, it was calculated by taking into account the arithmetic mean of the gold collected by the buyers (O’Neill & Telmer, 2017). The ratio (Hg/Au) is the amount of mercury used to produce a given amount of gold. It was determined by averaging the (Hg/Au) ratios in the site (O’Neill & Telmer, 2017). The amount of mercury used at the site per year [Q(Hg)/year] was deduced by multiplying the amount of gold produced by the ratio (Hg/Au). According to O’Neill and Telmer (2017), it is expressed by the following formula: *Q* (Hg)/year = *Q* (Au)/year × ratio (Hg/Au).

### Soil and water sampling and analysis

The soil and water samples were taken randomly to assess the level of mercury pollution. A total of 23 soil samples were taken at 0–20 cm depth including 14 soil samples taken from the washing sheds, 3 from the washing sludge area, 02 from the grinding mills, 02 from the gold purchasing sheds and 2 samples from the field, to assess potential pollution in the fields. In each area 3 soil samples have been taken and mixed to get a composite. The coordinates of the different sampling points were taken using a GPS. Two groundwater samples were taken from the two boreholes available that provide drinking water in the site.

In the laboratory, soil samples were dried, and sieved (0.5–2 mm) for chemical analysis. Organic matter (OM) content in soil was determined by the (Walkley & Black, 1934) method. The pH (H2O) was determined using an electronic pH meter in 1 g of soil in 2.5 mL of water. Water samples were preserved by adding 4 mL of conc. HNO3 (70%) to 1 L of sample and stored in a refrigerator at about 4 °C for subsequent analysis. One (1) g of soil sample was digested in 5 mL of HNO3 overnight before analysis. All the determinations were made in duplicate and quality control was done by using both certified material and recovery of mercury in soil samples, according to a previous paper (Olivero & Solano, 1998). Total mercury analysis (THg) in soil was determined by atomic absorption spectroscopy (AAS) after pyrolysis at 550 °C.

### Geo‑accumulation index (I_geo_)

The geo-accumulation (*I*_geo_) index is to evaluate contamination level in soils as given by (Müller, 1969; Loska et al., 1997).

$$\overline{\perp }geo=\mathit{Log}2\left(\frac{{C}_{Hg}}{1.5{B}_{Hg}}\right)$$

C_Hg_ is the measured concentration of Hg in soil, and *B*_*Hg*_ is the background concentration of the same metal.

### Statistical analysis

Microsoft Excel was used for calculating the workforce, averages, standard deviation and coefficient of variation. The statistical data processing was done using SPSS Statistics 20 and Microsoft Excel 2016 software. The SPSS Statistics 20 software was used to correlate the mode of mercury use with the social characteristics of individuals at the Gnikpière site, as well as the impact of pH and OM on the mercury content in soil.

## Results and discussion

### Population experience on artisanal gold mining in the Gnikpière site

According to the survey, a total of 2252 people were directly related to gold mining activities (50.02%) and 2248 (49.98%) were indirectly involved in gold mining. This population was composed of 62.5% of men and 37.5% of women (Table 1).

**Table 1:** Estimation of the workers in the study site

	Activities	Number of people	Percentage
Activities directly related to gold mining	Digging	800	17.78
	Crushing/grinding	150	3.33
	Washing the ore	1200	26.67
	Cyanidation of the ore	102	2.27
Other	Food sellers	400	8.89
	General trade	1000	22.22
	Motorcycle mechanics	80	1.7
	Other	768	17.06
Proportion (%)			
Women			37.5
Men			62.5

The mean durations of their activity in gold mining were 10 years, ranging from 2 to 25, and the mean durations of their activity in mercury use were 8 years, ranging from 2 to 21 (Table 2).

**Table 2:** Experience in artisanal mining and mercury use

	Average (years)	Minimum (years)		Maximum (years)	SD
Experience in mining	10.58	2	25		5.406
Experience in mercury use	8.72	2	21		4.894

The low average of years of experience in mercury use among the population of the Gnikpière site is due to the young population and the fact that the gold content has significantly decreased over the past 2 years. Many miners are working illegally and the mobility of miners is very high; they are frequently moving from one mining site to another where they believe they can find more extractable gold (Sousa & Veiga, 2009). The majority of experienced mining artisans have joined other promising sites within the country and in neighboring countries. This lack of experience among the mining artisans could contribute to an inefficient use of mercury in amalgamation and, consequently, to an increase in mercury pollution (Ottenbros et al., 2019).

### Mercury use in the study site

Amalgamation can be done with the whole ore coming from the crusher or with the amalgamation of a gravity concentrate. On the Gnikpière site, 100% of the surveyed artisanal gold miners perform amalgamation of a gravity concentrate, and no cases of amalgamation with whole ore have been reported or seen (Table 3). Unlike our study site, several sites in Burkina Faso still practice the amalgamation with whole ore. This situation is the case for the Warweogo, Wonghin, and Youga sites in the Center-East, the Mousso site in the Hauts-Bassins region, and the Sigtenga site in the North (AGC, 2019). Furthermore, 100% of the artisanal gold miners mix the gravity concentrate with their hands without protection. For amalgam burning, the artisanal gold miners use different energy sources. Indeed, the most commonly used energy sources are the furnace (35%), gas (10%), a fire pit (10%), or a combination of the mentioned methods (40%). Only 5% of mining artisans use blowtorches with retorts. Amalgamation and burning are activities practiced all over the Gnikpière site by artisanal gold miners. If some practices have been done in different places (32.5%), amalgamate and burn are being done in the same place by 67.5% of miners. All the operations on the site are done in the open areas.

**Table 3:** Mode of mercury use in the study site

		Percentage of miners (%)
Amalgamation	Amalgamation of the whole ore Amalgamation of a gravity concentrate	00 100
Amalgamation method	By hand Other method	100 00
Amalgamation burning method	Wood or butane fireplace Propane or gasoline torch	95 05
Amalgamation and burning site	Same area for both activities Different areas for both activities	67.5 32.5
Management of mercury waste	Throw in the environment Other method	100 00

These practices have also been reported by Veiga et al. (2014). The mercury treatment process (amalgamation and burning) generates waste such as mercury packaging (plastic bags) and contaminated materials. To these practices, we must add the large piles of washing sludge stored at the sheds. The majority of artisanal gold miners tend to abandon these mercury-contaminated wastes in the environment (Table 3). The lack of equipment management used for mercury treatment is also reported by Veiga et al. (2014).

### Artisan gold miners’ awareness on mercury impacts on health and the environment

The awareness on the risks associated with the use of mercury at the Gnikpière site varies from one artisan gold miner to another. Indeed, 87.5% of the surveyed miners claim to be aware of the risks of mercury use on their health, compared to 12.5% who are unaware. Also, 75% say to be aware on the risks of mercury use on the environment compared to 25% who know nothing about it. If 42.5% admit having been made aware of the risks associated with the use of mercury on their health and the environment, the majority (57.5%) say they have never been made aware of this issue (Table 4).

**Table 4:** Knowledge and awareness of artisanal miners in the study site

	Knowledge on health risks of mercury (%)	Knowledge on environmental risks of mercury (%)	Awareness on risks of mercury (%)
Yes	87.5	75	42.5
No	12.5	25	57.5

However, in Tanzania, the interviews in artisanal gold mining revealed wide variations in knowledge and risk perceptions concerning mercury and arsenic exposure, with 40.6% not aware of the health effects of mercury. This lack of knowledge, combined with minimal environmental monitoring and controlled waste management practices, highlights the need for health education, surveillance, and policy changes (Charles et al., 2013).

At the Gnikpière site, the working conditions are deplorable because, during the amalgamation and burning operations, no protective equipment is used by the artisan gold miners. Indeed, 87.5% of the miners claim to be aware of the risks of mercury use on their health. These results showed how consciously the mining artisans are willing to destroy the environment and risk their lives at the expense of gold. For them, only God can decide the fate of humans, no matter what they do. However, only 42.5% have been made aware at least once of the risks associated with the use of mercury on health and the environment. This means that their knowledge of the risks is very low. The lack of awareness of the real dangers of mercury use would explain their negligence towards this scourge.

Unlike the Gnikpière site in Zougnazagmiline in the North-Central region, only 53% of the artisan gold miners were aware of the risks of mercury use on health and the environment (Roamba, 2014). This low rate was explained by the distrust of some miners at the time. Indeed, they were afraid of reprisals from the authorities that could have repercussions on their activities; that is not the same situation during our study.

### Mercury/gold ratio used in the study site

The mercury/gold ratio is the amount of mercury used to produce a given amount of gold. According to the survey results, the mercury/gold ratio at the Gnikpière mining site varies between 1 and 3.5, with an average ratio of 2.16 (Table 5).

**Table 5:** Ratio of mercury/gold used by miners in the study site

		Values
Ratio mercury/gold	Minimum	1
	Maximum	3.5
	Average	2.16
	SD	0.5357

The amount of mercury used at the Gnikpière mining site per year is deduced from the amount of gold produced and the ratio (Hg/gold). The results have shown that 709.56 kg of mercury are used per year on the site. The high (Hg/Au) ratio obtained at the Gnikpière site could be due to the low gold content of the ore and the inexperience of several miners involved in the amalgamation process. Moreover, this result is consistent with the average (Hg/Au) ratio in the Southwest region, which is 2.37, but significantly higher than the national average (Hg/Au) ratio, which is 1.57 (PAN, 2020). However, it is significantly higher than the target set by the National Action Plan for 2023, which is 0.78 (PAN, 2020). Moreover, other sites exhibit a similar (Hg/Au) ratio to that of the Gnikpière site. This is the case for the Pouya and Dossi sites in the Hauts-Bassins region, which have a ratio (Hg/Au) of 2.2 (AGC, 2019).

The amalgamation of a gravity concentrate practiced by almost all the artisanal miners at the Gnikpière site could be explained by the low concentration of the gold in this site. Indeed, when the gold content is low, practicing amalgamation of the whole ore requires high quantities of mercury, and this is costly for the miners. This practice is forbidden by the Minamata Convention, which stipulates in its Article 7, paragraph 3, that each State must take measures to eliminate the amalgamation of whole ore. However, open-air burning (5% use the retort) and near dwellings is among the worst practices to eliminate in the gold panning activities listed in Annex C of the convention. However, several sites in Burkina still practice the amalgamation of the whole ore. This is the case of the Warweogo site, Wonghin and Youga in the Center-East, the Mousso site in the Hauts-Bassins region and the Sigtenga site in the North (AGC, 2019).

The waste from amalgamation is abandoned in the environment, and this could be due to negligence by the miners.

This situation could be explained by the lack of training of miners on mercury use. Indeed, the quantity of mercury needed for amalgamation depends on the gold content and particle size in the ore. Therefore, without basic training, only experienced miners can use mercury efficiently.

Furthermore, among the four worst practices that need to be eliminated from EMAPE as stated during the Minamata Convention, only the amalgamation of raw ore is not practiced at the Gnikpière site. Thus, in addition to the misuse of mercury on the site, the equipment used is inadequate, and this could lead to environmental pollution.

The gold production at the Gnikpière site represents 3.81% of the artisanal gold production in the Southwest, estimated at 8605 kg/year (AGC, 2019). The amount of mercury used at this site depends on this production. It represents 3.47% of the mercury used in the region (20,393 kg/year) (AGC, 2019; PAN, 2020). These quantities of mercury used at the Gnikpière site could be explained by the absence of alternative methods for gold recovery and the inefficient use of mercury (Hg/Au ratio = 2.16) (Table 5).

According to a study by AGC (2019) as part of the initial estimates of the artisanal and small-scale gold mining sector in Burkina Faso, around 71% of the initial mercury is released into the environment, with 53% into the air during burning and 18% into the soil during amalgam production. An extrapolation of these results in the present study shows that approximately 678.72 kg of mercury is released into the environment each year, with 359.72 kg in the air and 115.72 kg in the soil. The results showed the extent of mercury use at the Gnikpière site and its impact on the environment and the health of the population since mercury is persistent in the environment (Beavis & McWilliam, 2018).

### Impacts of gold mining on soil characteristics and mercury concentration

The results showed that the pH of all the soil samples taken from the Gnikpière mining site ranges between 6.40 and 8.96. The soil pH at the amalgamation site is 7.49, 7.65 at the field and 7.84 at the counter. At the mills, the pH is 6.71 and the washing sludge pH is 8.08, which are respectively slightly acidic and alkaline (Table 6). The rate of organic matter (OM) in the soils varies between 0.42% and 1.70% in the sampled sites. The soils poor in OM are recorded at the level of washing sludge (average OM = 0.46%). However, the organic matter of the soil in the amalgamation sites is 1.218%, in gold buying counters is 1.025%, in mills is 1.421%, and in fields is 1.477% (Table 6).

**Table 6:** Impacts of gold mining on soil characteristics and mercury concentration

Sampling site		pH_H2O_	OM (%)	Hg (ppm) UCC: 0.05 DL: 0.03	*I*_geo_
Amalgamation site	Minimum	6.58	1.200	2.3	
	Maximum	8.96	1.697	56.1	
	Average	7.49	1.218	19.2	8
	SD	0.54	0.302	16.1	
	CV	7.20	24.79	84	
Washing mud	Minimum	7.50	0.417	20.2	
	Maximum	8.45	0.538	85.1	
	Average	8.08	0.467	52.6	9.42
	SD	0.50	0.063	32.4	
	CV	6.18	13.490	62	
Crushing site	Minimum	6.40	1.402	113.0	
	Maximum	7.03	1.441	163.9	
	Average	6.71	1.421	138.4	10.8
	SD	0.44	0.028	36	
	CV	6.55	1.970	26	
Gold buying counter	Minimum	7.43	0.843	43.5	
	Maximum	8.25	1.209	52.8	
	Average	7.84	1.025	48.1	9.03
	SD	0.57	0.258	6.5	
	CV	7.27	25.170	14	
Field	Minimum	7.64	1.412	2.8	
	Maximum	7.67	1.543	4.5	
	Average	7.65	1.477	3.6	5.58
	SD	0.02	0.092	1.2	
	CV	0.26	6.228	33	

*CV* = variation coefficient, *UCC* = mercury concentration in the rock, *DL* = detection limit

For the mercury concentration in the soil, the results (Table 6) showed that mercury is present in all the soil samples from the Gnikpière site. The levels range from 2.8 ppm to 163.9 ppm. The soils at the mills record the highest levels of mercury (138.45 ppm), followed by the washing sludge (52.6 ppm), gold buying sheds (48.15 ppm), washing sheds (19.27 ppm) and finally those taken from the fields downstream of the Gnikpière site which have the lowest mercury levels (3.65 ppm). The descriptive statistics of the data showed that only the amalgamation sites and the washing sludge had a coefficient of variation (CV) greater than or equal to 50%. These CV values indicate a more heterogeneous distribution of mercury in the sampling points.

A comparison of the mercury concentrations in these different soils and its average concentrations in the continental crust (UCC) taken as a reference in this study showed that all the samples have very high mercury levels compared to their average concentration in the Earth’s crust, which is 0.056 ppm (Wedepohl, 1995). However, the mercury levels in the fields, amalgamation sites, and gold buying counters are below the land cultivation standard, which is 50 ppm, except for the “amalgamation site 3” sample (56.1 ppm) and the “gold buying counter 2” sample (52.8 ppm), which slightly exceed it. According to the literature, the concentration of the mercury in the site depends on the site. Bandé et al. (2020) have found similar results in the Gold Panning Sites in the Northern Region (Burkina Faso). In this site the average value of mercury was 13.45 mg/kg of soil. However, Sawadogo et al. (2023) have found a concentration of 3.083 mg/kg of mercury in the soil and, 0.16 mg/kg of mercury in a soil in Ghana (Akoto et al., 2017).

At the mills and washing sludges, the mercury levels are significantly higher than the standard for land cultivation (Table 6). Regarding the environment in general, the contamination threshold used comes from the Regulation on the Protection and Rehabilitation of Land (RPRT) of the Quebec Ministry of the Environment. It sets the limit values for many contaminants in terms of environmental quality. For mercury, this value is 10 ppm.

The low concentrations of mercury in the fields could be explained by the low mobility of mercury in the soils and its volatilization, which could also decrease its concentration during its transport to points downstream from the Gnikpière site. Also, the fact that the fields are turned over each time and amendments are added could dilute the mercury content in these fields. Moreover, these fields benefit very little from the runoff water from the amalgamation and burning sites. Most of the water from these places goes into the sub-basin neighboring the one that contains the majority of the Gnikpière site, hence the low mercury levels in these fields. However, the plants can capture this mercury from the roots and transfer it into the food chain.

In the process of mercuration, 71% of the initial mercury is released into the environment, of which 53% in the air during the burning of the amalgam and 18% in the soil during the washing stage of the amalgamation (AGC, 2019). This low proportion from the in-situ soils explains the moderately low concentrations at the amalgamation sites. The mercury levels found at the gold buying counters are higher than those found at the amalgamation sites. This could be explained by the burning of amalgams at the gold buying counters, whereas the latter use retorts that retain the mercury fumes. After many operations, the retort is just shaken and the mercury falls to the soil. That makes these places more polluted than amalgamation sites where the amalgam is burned in the open air (furnaces and fire pits). In the same line the results from the study in soils around a gold mine, mainly in Bissa, a village in the commune of SABCÉ in Burkina Faso show that the mercury concentration is 3.83 mg/kg (Sawadogo et al., 2023). These concentrations are very low compared to those recorded at the Gnikpière site, and this could be explained by the low intensity of gold panning in Bissa at the time of the work.

However, the geo-accumulation (Igeo) index showed that all the samples were highly polluted with Igeo from 5.58 to 10. Similar results have been found by Sawadogo et al., (2023) in soils from Bissa gold mining where the soils samples from agriculture sites are classified from unpolluted to highly polluted.

### Mercury concentration in groundwater in the study site

On the Gnikpière site, the water used for drinking is groundwater. Indeed, two boreholes serve the Gnikpière site, with the first located on the site and the second in the village located downstream from the site (300 m) and near the outlet of the subwatershed. The analysis of water samples taken from these two boreholes did not detect any content of mercury. Therefore, these waters meet the WHO standard adopted by Burkina Faso for mercury, which is 0.5 µg/L (Table 7). The absence of mercury in the water samples could be due to time, the type of aquifer, and the percolation rate of mercury. Indeed, with the Gnikpière site being located in an area of fractured aquifers, surface pollution would take several years to reach the water table, gold exploitation began at the Gnikpière site only 5 years ago. Moreover, mercury is a weakly mobile compound in soils, as it is generally mobilized by organic matter (Thomassin et al., 2003). These results are similar to those from Kouadio in 2016 on the borehole and well waters of the Galgouli site in southwestern Burkina Faso. At the Zougnazagmiline site in 2014, the mercury levels exceeded the WHO standard in borehole water samples located upstream and downstream of the gold mining site (Roamba, 2014). For the author, the presence of mercury in the groundwater samples could be due to the intensity of gold panning at the site and an influx from upstream sites.

**Table 7:** Mercury concentration in groundwater in the study site

Samples	Hg (µg/L)
Groundwater 1 Groundwater 1	< DL < DL

*DL* = Detection Limit = 0.5 µg/L

## Conclusion

Artisanal gold mining plays an important role in the economies of West African countries and particularly for local populations in Burkina Faso. However, the techniques used to extract gold can be dangerous for human health and the environment.

The study revealed that the amalgamation of concentrated gold is practiced by all the artisanal gold miners.

The groundwater of the Gnikpière site is free of mercury while the soil samples are all contaminated by mercury. The majority of mining artisans are aware of the risks of using mercury on their health and the environment, but many of them have never received any awareness training on its harmful effects. Due to the presence of mercury in the soil, investigations are needed to better estimate the extension of the pollution in order to limit its consequences. Considering the level of awareness of the artisanal gold miners, the government of Burkina Faso should sensitize miners on the health and environmental risk of mercury use.

## Data Availability

No datasets were generated or analyzed during the current study.

## Declarations

All authors have read, understood, and have complied as applicable with the statement on “Ethical responsibilities of Authors” as found in the Instructions for Authors.
